# Supplemental carnitine affects the microRNA expression profile in skeletal muscle of obese Zucker rats

**DOI:** 10.1186/1471-2164-15-512

**Published:** 2014-06-21

**Authors:** Janine Keller, Robert Ringseis, Klaus Eder

**Affiliations:** Institute of Animal Nutrition and Nutritional Physiology, Justus-Liebig-University, Heinrich-Buff-Ring 26-32, Giessen, 35392 Germany

**Keywords:** Carnitine, microRNA expression profile, Microarray, Skeletal muscle, Obese Zucker rat

## Abstract

**Background:**

In the past, numerous studies revealed that supplementation with carnitine has multiple effects on performance characteristics and gene expression in livestock and model animals. The molecular mechanisms underlying these observations are still largely unknown. Increasing evidence suggests that microRNAs (miRNAs), a class of small non-coding RNA molecules, play an important role in post-transcriptional regulation of gene expression and thereby influencing several physiological and pathological processes. Based on these findings, the aim of the present study was to investigate the influence of carnitine supplementation on the miRNA expression profile in skeletal muscle of obese Zucker rats using miRNA microarray analysis.

**Results:**

Obese Zucker rats supplemented with carnitine had higher concentrations of total carnitine in plasma and muscle than obese control rats (P < 0.05). miRNA expression profiling in skeletal muscle revealed a subset of 152 miRNAs out of the total number of miRNAs analysed (259) were identified to be differentially regulated (adjusted P-value < 0.05) by carnitine supplementation. Compared to the obese control group, 111 miRNAs were up-regulated and 41 down-regulated by carnitine supplementation (adjusted P-value < 0.05). 14 of these miRNAs showed a log2 ratio ≥ 0.5 and 7 miRNAs showed a log2 ratio ≤ −0.5 (adjusted P-value < 0.05). After confirmation by qRT-PCR, 11 miRNAs were found to be up-regulated and 6 miRNAs were down-regulated by carnitine supplementation (P < 0.05). Furthermore, a total of 1,446 target genes within the validated miRNAs were revealed using combined three bioinformatic algorithms. Analysis of Gene Ontology (GO) categories and KEGG pathways of the predicted targets revealed that carnitine supplementation regulates miRNAs that target a large set of genes involved in protein-localization and -transport, regulation of transcription and RNA metabolic processes, as well as genes involved in several signal transduction pathways, like ubiquitin-mediated proteolysis and longterm depression, are targeted by the miRNAs regulated by carnitine supplementation.

**Conclusion:**

The present study shows for the first time that supplementation of carnitine affects a large set of miRNAs in skeletal muscle of obese Zucker rats suggesting a novel mechanism through which carnitine exerts its multiple effects on gene expression, which were observed during the past.

**Electronic supplementary material:**

The online version of this article (doi:10.1186/1471-2164-15-512) contains supplementary material, which is available to authorized users.

## Background

Carnitine is a metabolite which is best known for its role as a shuttling molecule for the transport of long-chain fatty acids from the cytosol into the mitochondrial matrix where β-oxidation of fatty acids occurs [1]. Besides this essential function in intermediary metabolism, extensive studies over the past years revealed that supplementation with carnitine has positive effects on performance characteristics (e.g. growth rate, reproductive performance, protein:fat accretion) in livestock animals [2–6]. In addition, convincing evidence has been provided that supplemental carnitine is useful for the treatment of metabolic disorders that are associated with an impaired glucose tolerance and/or insulin sensitivity like diabetes and insulin resistance both, in model animals and in humans reviewed in [7]. With regard to the mechanisms underlying the beneficial effects of carnitine it has been shown that carnitine supplementation alters the expression of a large number of genes involved in important metabolic pathways, like glucose uptake and oxidation, fatty acid oxidation and protein degradation as well as critical signalling pathways, like the IGF-1/PI3K/Akt pathway, in liver and/or skeletal muscle [8–11], which may explain at least partially the abovementioned effects in livestock and model animals and humans. However, the exact molecular mechanisms how supplemental carnitine alters gene expression in tissues are largely unknown.

Increasing evidence suggests that microRNAs (miRNAs), a class of small non-coding RNA molecules (~22 nucleotides), play an important role in post-transcriptional regulation of gene expression and thereby influence a large number of physiological processes including differentiation, proliferation, apoptosis and immune response, but also metabolic processes, like glucose and lipid metabolism [12–14]. After maturation of the initially transcribed, so-called primary miRNAs to the single-stranded mature form, miRNAs are loaded into the RNA-induced silencing complex (RISC), which subsequently interacts with the miRNA recognition element (MRE) especially in the 3’ untranslated region (3’UTR) of the corresponding mRNA (target mRNA) [13, 15–17]. The kind of binding and the involved proteins determine the further fate of the target mRNAs, whose translation is either repressed or which are degraded [18, 19], thereby leading to down-regulation of gene expression. Less commonly, miRNAs can also up-regulate gene expression [20]. Noteworthy, single miRNAs can regulate the expression of hundreds of protein coding target mRNAs and, conversely, the expression of a single gene can be regulated by multiple miRNAs, indicating the complexity of miRNA-mRNA interrelationship and the great regulatory potential of miRNAs [13, 21, 22].

Up to now, several experiments and clinical analysis have been published demonstrating an altered miRNA expression profile and a modified phenotype due to an altered translation of target mRNAs in response to different pathologies [23–26] or pharmacologic treatment [27]. In contrast, only few studies have evaluated the influence of dietary factors, such as high-cholesterol diet [28] or conjugated linoleic acids [29], on the miRNA expression profile and its impact on gene expression in model or livestock animals. In a recent study, we could demonstrate that carnitine supplementation has beneficial effects in obese Zucker rats [30] - an established genetic model of obesity, metabolic syndrome and diabetes. The beneficial effect of carnitine supplementation was evident by the observation that the obesity-induced impairment of carnitine status and metabolic disturbances (mitochondrial dysfunction, impaired fatty acid oxidation, elevated plasma levels of triacylglycerides and non-esterified fatty acids) were significantly improved or even normalized [30]. The normalization of metabolic disturbances in obese Zucker rats due to carnitine supplementation was accompanied by an up-regulation of genes involved in carnitine uptake, fatty acid transport and uptake, β-oxidation, glucose uptake and glycolysis, and a fiber switch from type II to type I in skeletal muscle compared to non-supplemented Zucker rats [30]. In light of the important role of miRNAs for regulating gene expression, it is likely that at least some of these profound changes in gene expression in obese Zucker rats due to carnitine supplementation were mediated by altering miRNA expression in skeletal muscle. To our knowledge it has not been studied yet whether dietary carnitine influences miRNA expression. Therefore, the aim of the present study was to investigate the hypothesis that carnitine supplementation influences the miRNA expression profile in skeletal muscle of obese Zucker rats by using a miRNA microarray.

## Results

### Growth performance

Initial and final body weights after 28 days as well as daily body weight gains did not differ between the obese control group (367 ± 11, 507 ± 18, and 5.00 ± 0.54 g, respectively) and the obese carnitine group (375 ± 18, 520 ± 19, and 5.18 ± 0.34 g, respectively) (mean ± SD, n = 6). Feed intake and feed conversion ratio also did not differ between the obese control group (25.6 ± 1.4 g/d and 5.14 ± 0.34 g feed/g body weight gain, respectively) and the obese carnitine group (26.4 ± 3.0 g/d and 5.12 ± 0.74 g feed/g body weight gain) (mean ± SD, n = 6).

### Carnitine concentrations in plasma and skeletal muscle

**Figure 1:**
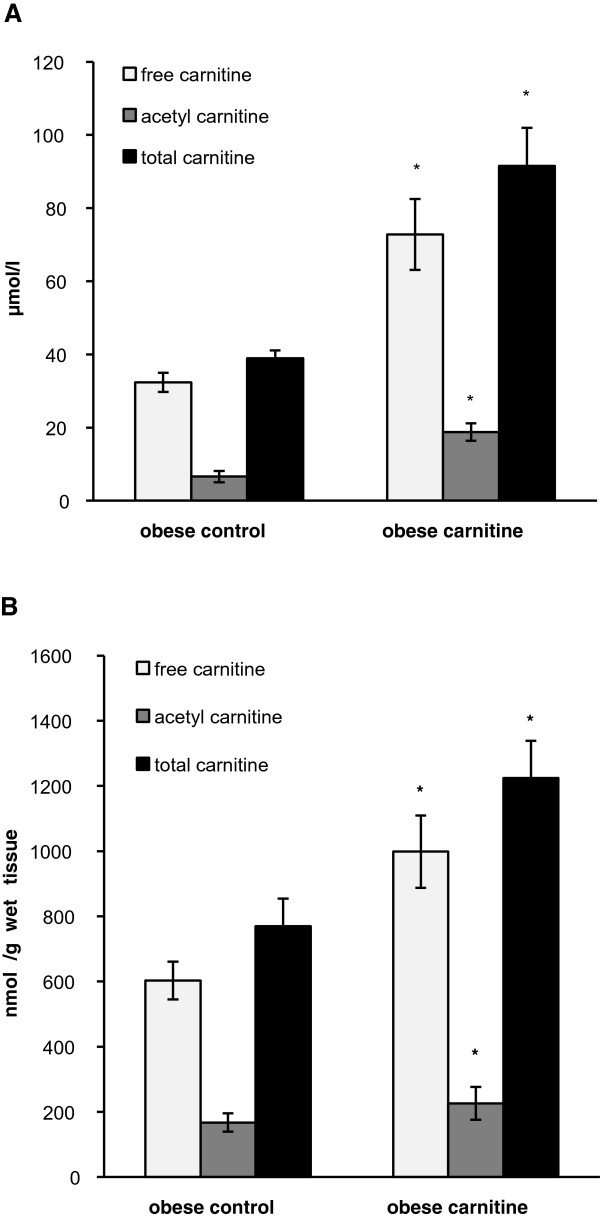
**Concentrations of carnitine in plasma and skeletal muscle of obese Zucker rats.** Concentrations of free, acetyl and total carnitine in plasma **(A)** and skeletal muscle **(B)** of obese Zucker rats fed either a control diet (obese control) or a diet supplemented with carnitine (obese carnitine). *Indicates significant difference from the obese control group (P < 0.05).

As shown in Figure 1, obese Zucker rats fed the diet supplemented with carnitine had higher concentrations of free-, acetyl- and total carnitine in plasma and skeletal muscle than those of the control group (P < 0.05).

### miRNA expression profiling in skeletal muscle of obese Zucker rats

**Figure 2:**
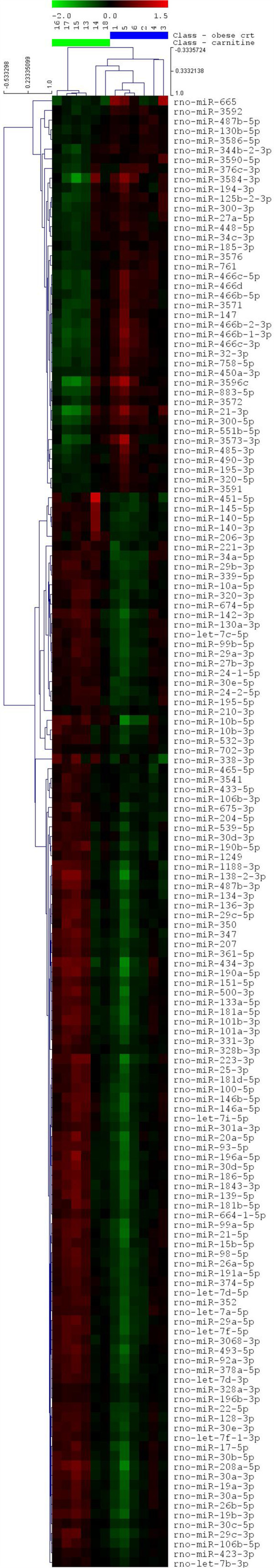
**Heat map and unsupervised hierarchical cluster analysis of the differentially expressed miRNAs in skeletal muscle of obese Zucker rats by supplemental carnitine.** The clustering was provided by Exiqon Services (Denmark) and carried out using the complete-linkage method together with the euclidean distance measure. Differentially expressed miRNAs chosen with an adjusted P-value < 0.05. Each row represents an individual miRNA and each column represents a sample. The miRNA clustering tree is shown on the left. The color scale illustrates the relative expression level of miRNAs. Red color represents an expression level below the reference channel, and green color represents expression higher than the reference. The codes on the legend are log2-transformed values.

**Figure 3:**
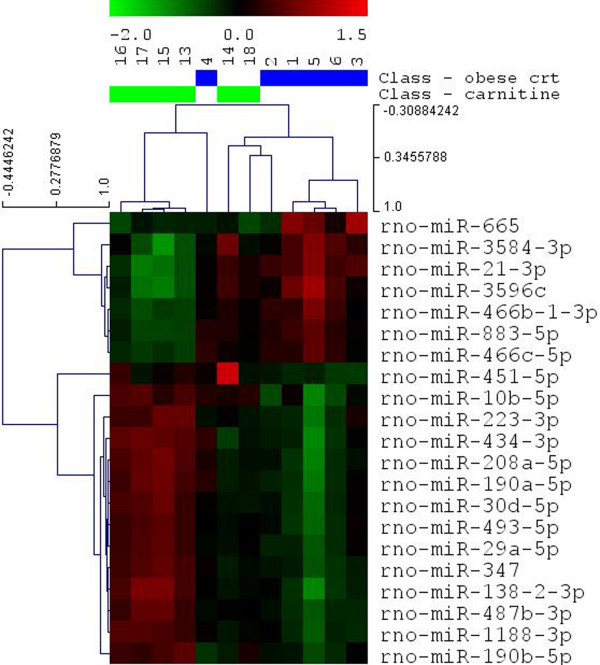
**Heat map and unsupervised hierarchical cluster analysis of the most strongly differentially expressed miRNAs in skeletal muscle of obese Zucker rats by supplemental carnitine.** The clustering was provided by Exiqon Services (Denmark) and carried out using the complete-linkage method together with the euclidean distance measure. Most strongly differentially expressed miRNAs chosen with a log2 Ratio ≥ 0.5 and ≤ −0.5 and an adjusted P-value < 0.05. Each row represents an individual miRNA and each column represents a sample. The miRNA clustering tree is shown on the left. The color scale illustrates the relative expression level of miRNAs. Red color represents an expression level below the reference channel, and green color represents expression higher than the reference. The codes on the legend are log2-transformed values.

To investigate the effect of supplemental carnitine on the muscle expression of miRNAs in obese Zucker rats, we conducted miRNA microarray analysis using miRCURY LNA™ microRNA Array (7th Gen). According to this miRNA profiling, 152 out of the total 259 miRNAs analyzed were identified to be differentially regulated (adjusted P-value < 0.05) by carnitine supplementation. Compared to the obese control group, 111 miRNAs were up-regulated and 41 down-regulated by carnitine supplementation (adjusted P-value < 0.05). Group-specific signal intensities are shown in Figure 2 and listed separately in Additional file 1: Table S1 with log2 ratios and fold changes. 14 of these miRNAs showed a log2 ratio ≥ 0.5 and 7 miRNAs showed a log2 ratio ≤ −0.5 (adjusted P-value < 0.05). A detailed list of these miRNAs and their respective log2 ratios and fold changes as well as adjusted P-values can be found in Table 1. The distribution of group specific signal intensities of these 21 differentially expressed miRNAs with adjusted P-value < 0.05 are shown in Figure 3.

**Table 1:** **Most strongly up-regulated (log2 Ratio ≥ 0.5) and down-regulated (log2 Ratio ≤ −0.5) miRNAs in skeletal muscle of Zucker rats by supplemental carnitine**

ProbeID	Annotation	Log2 Ratio	FC	P-value*
*Up-regulated miRNAs*				
148278	Rno-miR-138-2-3p	0.68	1.61	0.023
10925	Rno-miR-10b-5p	0.65	1.57	0.023
148261	Rno-miR-208a-5p	0.60	1.51	0.027
14285	Rno-miR-487b-3p	0.60	1.51	0.023
11024	Rno-miR-223-3p	0.59	1.51	0.023
11246	Rno-miR-434-3p	0.54	1.45	0.049
27536	Rno-miR-190a-5p	0.54	1.45	0.033
19596	Rno-miR-30d-5p	0.52	1.43	0.027
42763	Rno-miR-347	0.52	1.43	0.023
148059	Rno-miR-493-5p	0.51	1.43	0.023
145638	Rno-miR-29a-5p	0.51	1.43	0.023
42866	Rno-miR-451-5p	0.51	1.42	0.037
148417	Rno-miR-1188-3p	0.51	1.42	0.023
42472	Rno-miR-190b-5p	0.50	1.42	0.023
*Down-regulated miRNAs*				
42586	Rno-miR-466c-5p	−0.51	−1.42	0.023
42462	Rno-miR-883-5p	−0.51	−1.43	0.023
148483	Rno-miR-466b-1-3p	−0.55	−1.46	0.023
148583	Rno-miR-3584-3p	−0.62	−1.54	0.041
42770	Rno-miR-665	−0.64	−1.55	0.027
148139	Rno-miR-3596c	−0.73	−1.66	0.027
17896	Rno-miR-21-3p	−0.78	−1.72	0.023

*P-values have been corrected for multiple testing by the Benjamini and Hochberg adjustment method.

### Validation of the most differentially expressed miRNAs by qRT-PCR

To validate the microarray data, we performed qRT-PCR to quantify the expression of the 21 most differentially expressed miRNAs. As shown in Table 2, the expression patterns of these miRNAs detected by qRT-PCR were consistent with the microarray data. Among these 21 differentially expressed miRNAs, 17 (miR-10b-5p, miR-223-3p, miR-208a-5p, miR-434-3p, miR-190a-5p, miR-30d-5p, miR-347, miR-493-5p, miR-29a-5p, miR-451-5p, miR-190b-5p, miR-466c-5p, miR-883-5p, miR-466b-1-3p, miR-21-3p, miR-3596c, miR3584-3p) were proven significant (P < 0.05) by qRT-PCR, one (miR-487b-3p) had a tendency to be significant (P = 0.06), and three (miR-138-2-3p, miR-1188-3p, miR-665) were not confirmed to be significant (Table 2). Only the 17 significantly validated differentially expressed miRNAs, which were proven significant by qRT-PCR, were used for the subsequent target prediction and functional analysis.

**Table 2:** **Validation of microarray results using qRT-PCR**

	Mean fold changes		P-value*	
miRNAs	microarray	qRT-PCR	microarray	qRT-PCR
miR-138-2-3p	1.61	1.07	0.023	0.676
miR-10b-5p	1.57	1.58	0.023	0.023
miR-487b-3p	1.51	1.26	0.023	0.060
miR-223-3p	1.51	1.59	0.023	0.015
miR-208a-5p	1.51	4.26	0.027	0.001
miR-434-3p	1.45	1.85	0.049	0.017
miR-190a-5p	1.45	1.50	0.033	0.016
miR-30d-5p	1.43	1.53	0.027	0.035
miR-347	1.43	1.52	0.023	0.048
miR-493-5p	1.43	1.64	0.023	0.001
miR-29a-5p	1.43	1.49	0.023	0.047
miR-451-5p	1.42	1.63	0.037	0.034
miR-1188-3p	1.42	1.12	0.023	0.500
miR-190b-5p	1.42	1.74	0.023	0.021
miR-466c-5p	−1.42	−1.49	0.023	0.021
miR-883-5p	−1.43	−2.28	0.023	0.005
miR-466b-1-3p	−1.46	−1.76	0.023	0.011
miR-21-3p	−1.72	−2.04	0.023	0.001
miR-3596c	−1.66	−2.33	0.027	0.004
miR-665	−1.55	−1.22	0.027	0.140
miR-3584-3p	−1.54	−2.78	0.041	0.003

*P-values have been corrected for multiple testing by the Benjamini and Hochberg adjustment method.

### Target prediction of the significantly validated differentially expressed miRNAs and functional analysis

**Figure 4:**
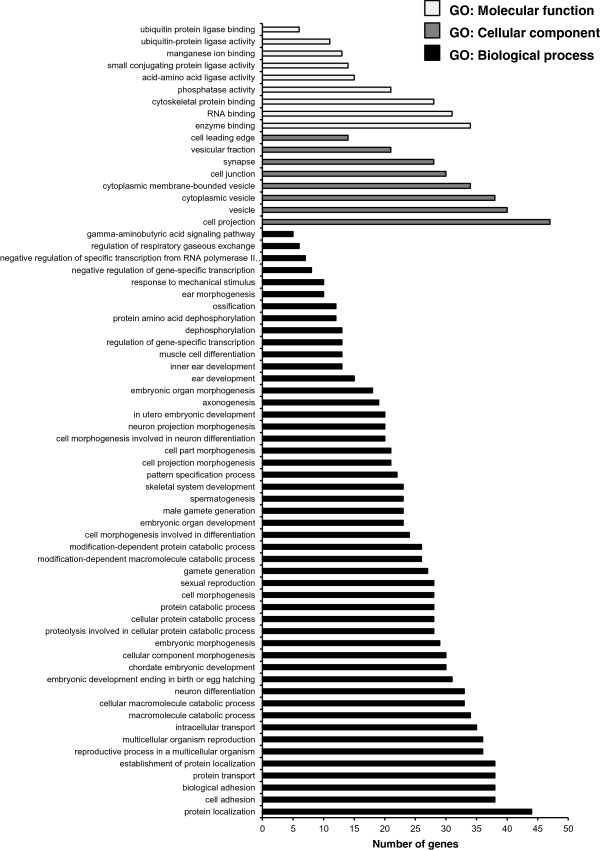
**Gene ontology analysis of the target genes of the validated 11 up-regulated miRNAs in skeletal muscle of Zucker rats with P < 0.01.** The GO terms were sorted by the number of genes in an ascending order from top to bottom.

**Figure 5:**
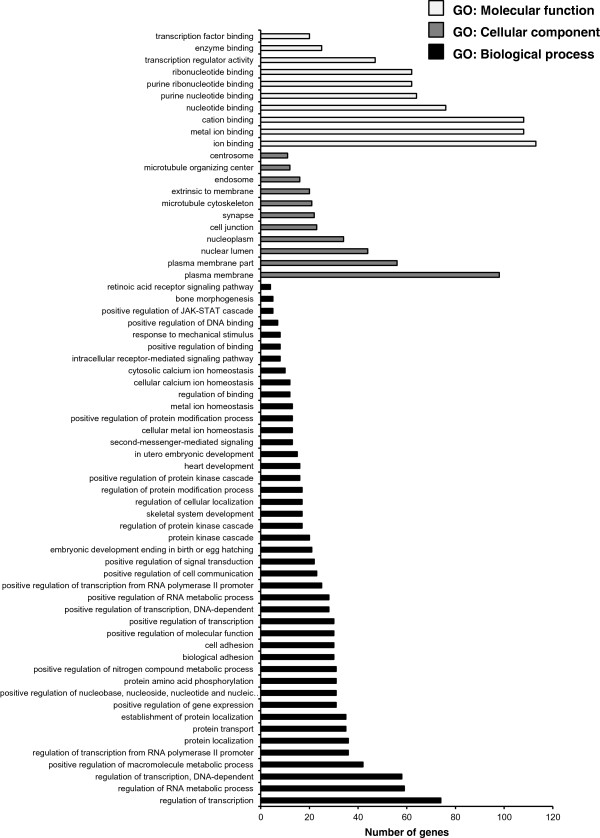
**Gene ontology analysis of the target genes of the validated 6 down-regulated miRNAs in skeletal muscle of Zucker rats with P < 0.01.** The GO terms were sorted by the number of genes in an ascending order from top to bottom.

We performed target prediction for the 17 significantly validated differentially expressed miRNAs (shown in Table 2) to identify the influence of carnitine supplementation on potential target mRNAs in skeletal muscle of obese Zucker rats by combining the results from three online free available algorithms. According to this, a total of 868 and 578 target genes were identified for the 11 up-regulated and 6 down-regulated miRNAs, respectively. Data are shown in Additional file 2: Table S2. To elucidate the biological functions of the predicted targets we carried out gene-term enrichment analysis using Gene Ontology (GO) categories and Kyoto Encyclopedia of Genes and Genomes (KEGG) pathway analysis by using the DAVID Functional Annotation Chart tool separately for the targets identified for the 11 up- and 6 down-regulated miRNAs.Gene-term enrichment analysis within GO category “biological process” revealed that most target genes predicted from the 11 up-regulated miRNAs were involved in protein localization, cell adhesion, biological adhesion, protein transport and establishment of protein localization (Figure 4, P < 0.01). In contrast, most target genes predicted from the 6 down-regulated miRNAs were involved in “biological processes” dealing with regulation of transcription, regulation of RNA metabolic process, as well as regulation of transcription, DNA dependent (Figure 5, P < 0.01). Within the GO category “molecular function”, most of the target genes predicted from the 11 up-regulated miRNAs were involved in enzyme- and RNA-binding and in cytoskeletal protein binding (Figure 4, P < 0.01), while most of the targets from the 6 down-regulated miRNAs had functions in ion-, metal ion- and cation-binding, as well as nucleotide binding (Figure 5, P < 0.01). Regarding the GO category “cell component”, the greatest number of targets predicted from the 11 up-regulated miRNAs had functions associated with cell projection (Figure 4, P < 0.01), whereas the functions of the targets predicted from the 6 down-regulated miRNAs were associated with plasma membrane (Figure 5, P < 0.01).

Gene-term enrichment analysis of the targets predicted from the significantly validated most differentially expressed miRNAs using KEGG pathways revealed several enriched regulatory pathways. As shown in Table 3, enriched KEGG pathways (EASE score < 0.05) for the 868 putative mRNAs from the 11 up-regulated miRNAs included pathways regulating cancer, the Wnt signalling pathway, ubiquitin mediated proteolysis, leukocyte transendothelial migration, adherens junction, longterm depression and ether lipid metabolism, and for the 578 putative targets from the 6 down-regulated miRNAs pathways regulating ubiquitin mediated proteolysis, leukocyte transendothelial migration, T cell receptor signalling and long-term depression (Table 3).

**Table 3:** **KEGG pathway analyses of the predicted target genes of the validated most differentially expressed microRNAs with P < 0.05**

Pathway	P-value	Genes
*Putative targets of the up-regulated miRNAs*		
Athways in cancer	0.044	WNT5A, TCF7, FGFR3, RALBP1, VHL, TGFBR1, IGF1, CTNNA1, STK4, ITGB1, IGF1R, CBLB, CCND1, HIF1A, CDKN1B, JUN, SOS2, HHIP, WNT9A, PIAS1
Wnt signalling pathway	0.012	WNT5A, TBL1XR1, TCF7, ROCK2, PPP2R5D, DKK2, CCND1, SIAH1A, JUN, CAMK2B, WNT9A, PPP3CA, FBXW11
Ubiquitin mediated proteolysis	0.011	CUL3, UBE2N, UBE2A, CBLB, UBE3A, VHL, UBE2K, SIAH1A, UBE2I, HERC2, PIAS1, FBXW11
Leukocyte transendothelial migration	0.037	GNAI2, ACTN4, ROCK2, CXCR4, RAP1A, RAPGEF4, CTNNA1, ITGB1, CLDN23, PTPN11
Adherens junction	0.026	IGF1R, PVRL4, TCF7, TJP1, ACTN4, TGFBR1, CTNNA1, SNAI1
Longterm depression	0.048	IGF1R, GNAI2, PLA2G12A, GRID2, IGF1, PRKG2, ITPR2
Ether lipid metabolism	0.044	PLA2G12A, PLA2G7, PAFAH1B1, PPAP2A, PPAP2B
*Putative targets of the down-regulated miRNAs*		
Ubiquitin mediated proteolysis	0.003	CUL3, UBE2N, UBE3A, VHL, RHOBTB2, BIRC6, UBA6, UBE2I, SMURF2, UBE2D1, CUL1
Leukocyte transendothelial migration	0.017	CLDN8, ITK, CLDN19, PIK3CA, RAPGEF4, CLDN11, ITGB1, MLLT4, PRKCB
T cell receptor signalling pathway	0.036	ITK, JUN, PIK3CA, CHP, DDAH1, NFATC3, LCP2, IL2
Long-term depression	0.042	GNAO1, IGF1, GUCY1B3, GRM1, ITPR1, PRKCB

### Validation of selected predicted target genes by qRT-PCR

To explore whether the differentially expressed miRNAs were associated with changes in the mRNA levels of respective target genes, we determined relative mRNA levels of one selected potential target gene for each validated miRNA by qRT-PCR. As shown in Table 4, the relative mRNA levels of predicted targets (UBE2A, HERC2, ARPC5, GNAI2, SLC6A8, IGF-1, ITPR1, ACSL3, and SALL3) of 9 differentially regulated miRNAs were significantly regulated by carnitine in the opposite direction (P < 0.05), whereas the relative mRNA levels of the predicted targets of the remaining differentially regulated miRNAs were not significantly inversely regulated.

**Table 4:** **Validation of predicted target-mRNAs using qRT-PCR**

Predicted target mRNAs	Related miRNA	Obese control	Obese carnitine
		Fold of control	
	*Up-regulated miRNAs*		
WNT5A	miR-10b-5p	1.00 ± 0.57	0.80 ± 0.46
UBE2A	miR-223-3p	1.00 ± 0.48	0.52 ± 0.10^*^
HERC2	miR-208a-5p	1.00 ± 0.07	0.87 ± 0.08^*^
ALCAM	miR-434-3p	1.00 ± 0.13	0.97 ± 0.16
ARPC5	miR-190a-5p	1.00 ± 0.18	0.73 ± 0.14^*^
GNAI2	miR-30d-5p	1.00 ± 0.14	0.72 ± 0.07^*^
FADS1	miR-347	1.00 ± 0.44	0.86 ± 0.11
PIAS1	miR-493-5p	1.00 ± 0.50	1.02 ± 0.59
CBLB	miR-29a-5p	1.00 ± 0.46	0.91 ± 0.25
SAMD4B	miR-451-5p	1.00 ± 0.38	0.98 ± 0.27
WSB1	miR-190b-5p	1.00 ± 0.41	0.67 ± 0.22
	*Down-regulated miRNAs*		
SLC6A8	miR-466c-5p	1.00 ± 0.44	2.34 ± 0.52^*^
ABCG1	miR-883-5p	1.00 ± 0.38	1.08 ± 0.26
IGF-1	miR-466b-1-3p	1.00 ± 0.28	2.26 ± 0.65^*^
ITPR1	miR-21-3p	1.00 ± 0.29	1.37 ± 0.15^*^
ACSL3	miR-3596c	1.00 ± 0.49	2.11 ± 0.87^*^
SALL3	miR-3584-3p	1.00 ± 0.44	6.32 ± 2.47^*^

Data are expressed as means ± SD and were presented as fold of the obese control group, which mean was set to 1; n = 6 rats/group. *Indicates significant difference to the obese control group (P < 0.05).

### IGF-1 protein level in skeletal muscle

**Figure 6:**
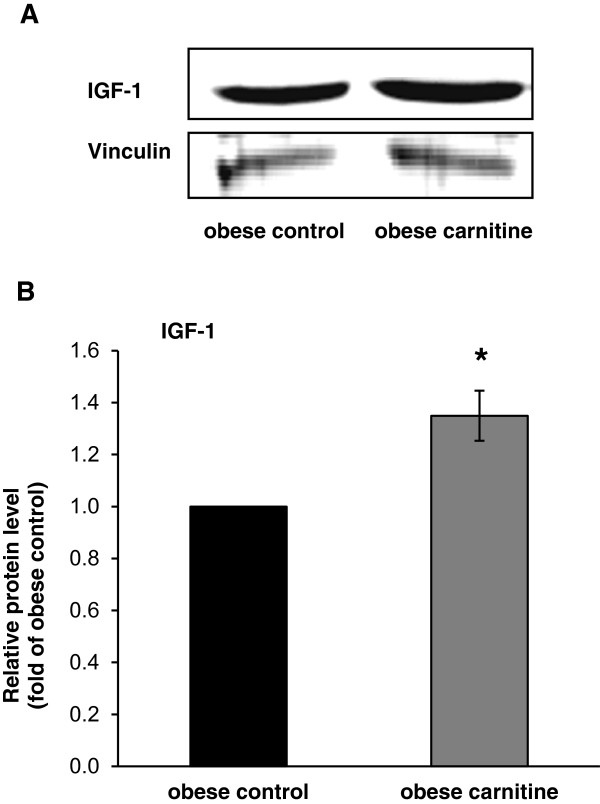
**Relative protein level of IGF-1 in skeletal muscle of obese Zucker rats. (A)** Representative immunoblots specific to IGF-1 and Vinculin as internal control are shown for one animal per group; immunoblots for the other animals revealed similar results. **(B)** Bars represent data from densitometric analysis and represent means ± SD (n = 6/group); bars are expressed relative to the protein level of the obese control group (=1.00). *Indicates significant difference from the obese control group (P < 0.05).

To further investigate whether the carnitine-induced increase in the mRNA level of IGF-1, a putative target gene of miR-466b-1-3p but also miR-208a-5p, is also associated with an increase in the protein level of IGF-1, we determined protein levels of IGF-1 in skeletal muscle of the obese Zucker rats. Consistent with the higher mRNA level of IGF-1, the relative protein level of IGF-1 was increased by 35% in the obese carnitine group compared to the obese control group (Figure 6, P < 0.05).

## Discussion

This is the first study demonstrating the response of the miRNA expression profile to dietary supplementation with carnitine. miRNAs represent a relatively newly identified class of small non-coding RNA molecules which play an important role for gene expression by mainly down-regulating the expression of protein-coding genes, and, consequently influence both, physiological and pathological processes. Since the molecular mechanisms underlying changes in gene expression by carnitine supplementation in livestock and model animals are only poorly understood, the present study aimed to elucidate possible alterations in the miRNA profile following dietary supplementation with carnitine. For miRNA profiling, we used skeletal muscle samples from carnitine-supplemented and control (non-supplemented) obese Zucker rats, in which we have recently reported profound changes in the expression of genes involved in important metabolic pathways, like glucose and fatty acid metabolism and protein degradation [30]. It is well known that the carnitine status in obese Zucker rats and other models of genetic and diet-induced obesity is markedly impaired and as a consequence several metabolic disturbances are induced [31–33]. In contrast, supplementation with carnitine improves carnitine status, which was also demonstrated in the present study, and reverses to a great part these metabolic disturbances, like elevated blood levels of triacylglycerides and non-esterified fatty acids, glucose intolerance and insulin resistance [30–32, 34]. The key finding of the present study is that carnitine supplementation resulted in a profound change in the miRNA expression profile in skeletal muscle of obese Zucker rats suggesting a novel regulatory mechanism by which dietary carnitine alters gene expression and mediates at least some of its biological effects. According to our miRNA microarray analysis, a total of 152 miRNAs were identified to be differentially expressed, 111 being up-regulated and 41 being down-regulated, in skeletal muscle of the obese Zucker rats in response to carnitine supplementation. In order to elucidate the functional implications of alterations in the miRNA profile it is a widely accepted approach to identify the miRNA target genes by employing specific bioinformatics tools. Because one single miRNA can regulate the expression of hundreds of target mRNAs, while one gene may be targeted by many miRNAs, target prediction is usually carried out only in a small subset of the differentially expressed miRNAs, typically in the most strongly differentially regulated miRNAs. Following this approach, we selected those miRNAs, which were significantly differentially expressed between the two groups at a log2 ratio of ≥ 0.5 and ≤ −0.5 according to microarray analysis and according to qRT-PCR, resulting in 11 up-regulated and 6 down-regulated miRNAs, respectively. The subsequent *in silico*-target mRNA prediction revealed 868 and 578 target genes for the 11 up-regulated and the 6 down-regulated miRNAs, respectively. Due to the limited conclusiveness of *in silico*-predictions, we determined mRNA levels of one individual target gene for each of the carnitine-regulated miRNAs using qRT-PCR in order to provide some proof of concept that predicted targets are indeed regulated in the expected direction *in vivo*. According to this, we observed that the majority of predicted target mRNAs of the differentially regulated miRNAs were regulated by carnitine in the opposite direction indicating that the predictions from *in silico*-analysis are reliable at least in some instances. One of the predicted miRNA-mRNA interactions, which could be confirmed *in vivo*, concerns the insulin-like growth factor (IGF)-1. IGF-1 is a known target of the down-regulated miR-466b-1-3p and has been demonstrated to be up-regulated by carnitine in the present study but also in previous studies [11, 35]. In order to strengthen the biological relevance of miRNA-mRNA interactions for regulating gene expression by carnitine in the case of IGF-1, we also determined protein levels of IGF-1. In line with the elevated mRNA level of IGF-1 in skeletal muscle, we also observed increased protein levels of IGF-1 in skeletal muscle of rats of the carnitine group. This suggests that the elevated mRNA level of IGF-1 is a result of a decreased repressive activity of the down-regulated miR-466b-1-3p and that miRNA-mRNA interactions indeed play a significant role in mediating biological effects of carnitine. One important reason that might explain that the predictions of target mRNAs for differentially regulated miRNAs are not reliable in all instances is that one gene is normally subject to regulation by a cluster of miRNAs, from which certain miRNAs can be regulated in the opposite direction. Another reason might be that the carnitine-mediated change in the expression of certain miRNAs was not strong enough to induce a significant change in the expression of the targeted mRNAs. Moreover, post-transcriptional modifications by carnitine might also be responsible for the observation that predicted miRNA-mRNA interactions are not visible *in vivo*. Finally, we carried out gene-term enrichment analysis within the three GO categories and the KEGG pathway database using DAVID software, separately for the targets identified from the prediction of the 11 up- and 6 down-regulated miRNAs in order to identify biological functions of the predicted targets.

Noteworthy, gene-term enrichment analysis of the targets predicted from the differentially regulated miRNAs revealed that carnitine supplementation regulates miRNAs that target a large set of genes involved in enzyme- and RNA-binding, regulation of transcription, regulation of transcription-DNA dependent, regulation of RNA metabolic process, and nucleotide-binding. This clearly indicates that carnitine supplementation regulates many genes which are involved in controlling gene expression and that gene regulation by carnitine supplementation is more complex due to miRNA-mRNA interactions than previously thought. Amongst the predicted target mRNAs of the regulated miRNAs we found about 60 mRNAs encoding transcription factors, which were classified according to their function into general and specific transcription factors (not shown). We found that the main part belonged to the class specific transcription factors, like ATF3, BCL6, BCOR, BHLHE23, BHLHE41, CREM, E2F6, ELK4, HBP1, HIVEP1, IRF9, JUN, KLF15, MEF2C and many others. These specific transcription factors usually bind upstream of the initiation site to specific recognition sequences of the regulated gene to stimulate or repress its transcription. Although it is difficult to directly relate the functions of most these specific transcription factors to recently observed carnitine effects in skeletal muscle, it is likely that carnitine mediates at least some of its effects on gene expression through regulating transcription factor activities by altering miRNA expression. The observation that the minor part of the transcription factors targeted by the carnitine-regulated miRNAs were general transcription factors, like GTF2B, GTF2I, HMGB2, MED14, MED17, and PAF1, does not exclude a significant involvement in inducing carnitine effects in skeletal muscle [7], because general transcription factors are essential for gene transcription to occur due to their obligatory role in transcription initiation by RNA polymerase II. Analysis of the involvement of the predicted target genes in specific KEGG pathways showed that particularly genes playing roles in signal transduction pathways, like the Wnt signalling pathway (e.g. WNT5A, TBL1XR1, TCF7, JUN, DKK2), long-term depression (e.g. IGF1, IGF1R, GNAI2 and PRKCB), and protein-degrading pathways, like ubiquitin-mediated proteolysis (e.g. UBE2A, UBE3A, VHL, UBA6, SMURF2), are targeted by the miRNAs regulated by carnitine supplementation. Regulation of miRNAs targeting genes belonging to ubiqutin-mediated proteolysis may provide a plausible explanation for our recent observations in rats and pigs that carnitine supplementation down-regulates key genes involved in ubiquitin-mediated proteolysis in skeletal muscle [9–11]. Ubiquitin-mediated proteolysis within proteasomes is the most important protein degradation pathway in tissues in general, and particularly responsible for the degradation of myofibrillar proteins in skeletal muscle which make up about 60% of total muscle proteins. Thus, the observation that carnitine supplementation reduces muscle wasting in a rat model of cancer cachexia [36], which is characterized by an increased activity of ubiquitin-mediated proteolysis, is largely explained by inhibition of this pathway. Inhibition of this pathway by carnitine supplementation is probably mediated by increasing plasma concentrations of IGF-1, which has been observed in several species including humans, pigs, rats and chickens [35, 37–41]. IFG-1 is an important negative regulator of this protein degrading pathway and this effect is mediated by an activation of the PI3/Akt signalling pathway leading to inactivation of FoxO transcription factors and thereby inhibition of the ubiquitin-ligating E3 ligases [42]. Recently, we have indeed observed that carnitine supplementation activates the IGF-1/PI3K/Akt signalling pathway, inactivates FoxOs, reduces expression of E3 ligases and decreases the amount of ubiquitinated proteins which are targeted for degradation via the proteasome [11]. Moreover, since IGF-1 has also prominent functions in cell proliferation and differentiation it was not surprising that enrichment analysis identified IGF-1 as one gene involved in the KEGG pathways “pathways in cancer” but also in “long-term depression”, that was targeted by the miRNAs down-regulated by carnitine supplementation. Thus, our observations indicate that the recently observed increases in mRNA and/or protein levels of IGF-1 following carnitine supplementation is mediated at the level of miRNA-mRNA interactions.

The large number of target genes (about 1,500) predicted from the most strongly regulated miRNAs and the other not explicitly mentioned data from gene-term enrichment analysis for the targets identified shows that carnitine supplementation may cause several other biological effects than up-regulation of IGF-1 or inhibition of ubiquitin-mediated proteolysis via miRNA-mRNA interactions. However, the biological implications of most of the observed carnitine-mediated changes in the miRNA expression profile and its impact on specific metabolic and signalling pathways and whole metabolism cannot be resolved with certainty using biostatistics tools alone, because the prediction of target genes from differentially expressed miRNAs is only an *in silico*-approach and, thus, has clear limitations. Therefore, time-consuming experimental studies with cultured muscle cells, in which the effect of carnitine in the presence of miRNA-specific inhibitors or during over-expression or knockdown of specific miRNAs on target gene expression is studied, are necessary in the future to validate at least some of the carnitine-mediated miRNA-mRNA interactions. Given that most of the detectable miRNAs (152 out of 259) were regulated by carnitine, it would be interesting to investigate in a future time-course experiment whether there are early and late responding miRNAs which behave differently to carnitine supplementation.

## Conclusions

The present study clearly shows for the first time that a large set of miRNAs in skeletal muscle of obese Zucker rats are responsive to carnitine supplementation suggesting a novel mechanism through which carnitine exerts its multiple effects on gene expression. Using biostatistics tools at least some of the recently reported effects of carnitine supplementation on gene expression (IGF-1, E3 ligases) could be demonstrated to be likely mediated via miRNA-mRNA interactions. Although the biological implications of most of the observed carnitine-mediated changes in the miRNA expression profile cannot be predicted using this biostatistics approach alone and experimental validations of miRNA-mRNA interactions are necessary, our results indicate that carnitine supplementation exerts several other biological effects through altering the expression of miRNAs.

## Methods

### Animal experiment

The animal experiment was approved by the local Animal Care and Use Committee. For this study, we used plasma and muscle samples from 6 obese Zucker rats per group from a previous experiment [30]. In this experiment, 8 to 10 week old, male obese (*fa/fa*) Zucker rats (Crl:ZUC-*Lepr*^*fa*^; Charles River, France) were randomly divided in two groups (obese control group and obese carnitine group) and fed semi-purified diets according to the recommendations of the American Institute of Nutrition (AIN)-93G [43] *ad libitum* for 28 days. The treatment duration was based on several reports in the literature that carnitine supplementation improves carnitine status and causes profound changes in gene expression in different tissues within a few weeks [7]. The obese carnitine group received the same diet supplemented with 3 g carnitine/kg diet. Blood was collected and plasma obtained by centrifugation, and skeletal muscle (*M. rectus femoris*) was excised and immediately stored at −80°C. A detailed description of the animal experiment and sample collection can be found in our previous publication [30].

### Carnitine analysis

Concentrations of free carnitine and acetyl carnitine in plasma and muscle were determined by tandem mass spectrometry using deuterated carnitine-d_3_ as internal standard as described recently in detail [44]. Concentration of total carnitine was calculated as the sum of free and acetyl carnitine.

### RNA isolation

For miRNA microarray and qRT-PCR analysis total RNA, including small RNAs, was isolated from muscle samples using the Qiagen miRNeasy Mini Kit (Qiagen, Hilden, Germany), according to the manufacturer’s protocol. Afterwards, the concentration of the RNA was determined using an Infinite 200 M microplate reader and a nanoQuant Plate (both from Tecan, Männedorf, Switzerland) and its integrity was confirmed by agarose gel electrophoresis. Isolated RNA samples were immediately frozen and stored at −80°C.

### miRNA microarray analysis

All microarray analyses were conducted at Exiqon Services (Denmark). Sample total RNA quality was verified using an Agilent BioAnalyzer 2100 System. MiRCURY LNA™ microRNA Arrays (7th Gen) following the miRBASE release 18 (http://www.mirbase.org/) were used to analyze the expression profile of each sample (Exiqon, Denmark). Briefly, 750 ng total RNA from both sample and reference was labeled using the mercury LNA™ microRNA Hi-Power Labeling Kit, Hy3™/Hy5™ (Exiqon, Denmark) and were mixed pair-wise and hybridized according to the instruction manual using a Tecan HS4800™ hybridization station (Tecan, Austria). Afterwards, slides were scanned using the Agilent G2565BA Microarray scanner System (Agilent Technologies, Inc., USA) and image analysis was carried out using the ImaGene® 9 (mercury LNA™ microRNA Array Analysis Software, Exiqon, Denmark). The quantified signals were background corrected (Normexp with offset value 10, see [45]) and normalized using the global Lowess (LOcally WEighted Scatterplot Smoothing) regression algorithm. The signal values were filtered based on absent/present calls. miRNAs with present calls < 20% were removed from the final dataset used for the expression analysis. The microarray data related to all samples have been deposited in NCBI’s Gene Expression Omnibus public repository [46].

### qRT-PCR validation of differentially expressed miRNAs

qRT-PCR reactions were performed using a Rotor-Gene 2000 system (Corbett Research, Mortlake, Australia) to validate the expression of 21 miRNAs, which showed significant differences in their expression level with an adjusted P < 0.05 in the microarray experiment. For this end, 0.5 μg of total RNA (RNA templates were the same as used for microarray hybridizations) were reverse transcribed using the miScript II RT Kit (Qiagen, Hilden, Germany), according the manufacturer’s protocol and was stored in diluted (1:10) aliquots at −20°C. 2 μl cDNA, 2 μl each of miScript Universal Primer (10x) and miScript Primer Assay (10x) and 4 μl RNase free water were added to 10 μl QuantiTect SYBR Green PCR Master Mix (2x) (all from Qiagen, Hilden, Germany). The qRT-PCR cycle comprised of, 15-minute incubation at 95°C followed by 40 cycles of a three-stage temperature profile of 94°C for 15 sec and 55°C for 30 sec and final 70°C for 30 sec. The relative changes of each transcript were calculated by using the the 2^-ΔCt^ equation [47] with the expression level of U6 snRNA (U6 small nuclear RNA) as an internal control.

### Prediction of targets of differentially expressed microRNAs and functional analysis

Prediction of putative miRNA targets was performed by using three online free available algorithms TargetScan release version 6.2 (http://www.targetscan.org/), miRanda and miRDB (http://mirdb.org/miRDB/). To identify enriched (overrepresented) Gene Ontology (GO) terms and Kyoto Encyclopedia of Genes and Genomes (KEGG) pathways for the target genes of the differentially expressed miRNAs, we used the Database for Annotation, Visualization and Integrated Discovery (DAVID) gene annotation tool [48].

### qRT-PCR validation of selected potential target genes

qRT-PCR analysis of predicted target genes and reference genes as well as calculation of gene expression data was performed as described recently in detail [10]. The normalization factor was calculated as the geometric mean of expression data of the three most stable out of six tested potential reference genes (CANX, MDH1, ACTB, RPL13, TOP1, and ATP5B). The three most stable reference genes were (the stability score M as calculated by GeNorm is shown in brackets): CANX (0.025), TOP1 (0.030) and RPL13 (0.036). Means and SD were calculated from normalized expression data for samples of the same treatment group. The mean of the obese control group was set to 1 and mean and SD of the obese carnitine group were scaled proportionally. Features of gene-specific primer pairs are listed in Table 5.

**Table 5:** **Characteristics of specific primers used for validation of target-mRNAs by qRT-PCR**

Gene	Forward Primer (3’-5’)	Reverse Primer (3’-5’)	Product length (bp)	T _m_(°C)	NCBI GenBank
*Reference genes*					
ACTB	GACCTCTATGCCAACACAGT	CACCAATCCACACAGAGTAC	154	60	NM_031144.2
ATP5B	GCACCGTCAGAACTATTGCT	GAATTCAGGAGCCTCAGCAT	203	60	NM_134364.1
CANX	CCAGATGCAGATCTGAAGAC	CTGGGTCCTCAATTTCACGT	175	60	NM_172008.2
MDH1	CAGACAAAGAAGAGGTTGCC	CGTCAGGCAGTTTGTATTGG	206	60	NM_033235.1
RPL13	CTTAAATTGGCCACGCAGCT	CTTCTCAACGTCTTGCTCTG	198	60	NM_031101.1
TOP1	GAAGAACGCTATCCAGAAGG	GCTTTGGGACTCAGCTTCAT	137	60	NM_022615.1
*Target genes*					
ABCG1	GCCATCCCTGTCTTGCTCTT	TCCTCTCGGTCCAAGCCATA	143	56	NM_053502.1
ACSL3	GTAAAACTTGATTCCCGTTGAGA	GTGTCGCAGCCAGGATACA	307	60	NM_057107.1
ALCAM	TCGCTGACCCTCATCGTAGA	ATCGTCTGCCTCATCGTGTT	321	60	NM_031753.1
ARPC5	GGGATGTCGAAGAACACGGT	GTAGGCATGAGTCCACCTCG	142	60	NM_001025717.1
CBLB	TTGAAGGGTGAAGATGCTTTTGAT	ACTGGAGCCTGGAGGTTTTG	105	60	NM_133601.1
FADS1	CCACTACGCTGGTCAGGATG	AGCGCCTTATTCTTGGTGGG	144	60	NM_053445.2
GNAI2	GCCGAGCGCTCTAAGATGAT	TGCTTGACGATGGTGCTCTT	119	60	NM_031035.3
HERC2	CCTGACCACCGAGAGGAAAC	ACACCATCATCTGGATATCTGTT	102	60	NM_001107520.1
IGF-1	CCCGGGACGTACCAAAATGAGCG	ATGTCAGTGTGGCGCTGGGC	354	64	NM_001082477.2
ITPR1	ATGCCAGGAGGAAATGCGAA	CTCAGGGGTGGACTTGGTTC	250	60	NM_006236992.1
PIAS1	ATGACCTGCTGGACGAACTG	ACTGTCGTGGACAATCGGAC	355	60	NM_001106829.2
SALL3	CTCTTCTTGGTTTCCTAGGCGT	TCCGCCCACTTGAAGAACTC	130	60	NM_001108892.1
SAMD4B	TGTGGACCTCCCCTGCTTTG	GAGCAAAGGCACAGAAACCTG	199	60	NM_001107498.1
SLC6A8	GGTCCCCTGTCATCGAGTTC	GAGGACCACGTAGGGGAATG	197	60	NM_017348.2
UBE2A	TGTGGAAACCACAGGACAACT	CAGTCACGCCAGCTTTGTTC	327	60	NM_001013933.1
WNT5A	TCCGCAGTCCTGCTTTGAAT	CAAAGCCACTCCTGGGCTTA	156	60	NM_022631.1
WSB1	GAGTTCCCGGAATCAGACGG	CCGGAGCAAAAGCAACAGTC	163	60	NM_00104256.1

### Western Blotting

For western blot analysis, frozen muscle samples (30 mg) were homogenized in RIPA buffer (radioimmunoprecipitation assay buffer; 50 mM Tris, 150 mM NaCl, 10% glycerol, 0.1% SDS, 1% Triton X-100, 1 mM EDTA, 0.5% deoxycholate, 1% protease inhibitor mix; pH 7.5) using an Ultraturrax (IKA Werke GmbH, Staufen, Germany). The homogenate was centrifuged at 16,200 *g* (4°C) for 15 min. Protein concentrations were determined in the supernatants using the bicinchoninic acid protein assay kit (Interchim, Montluçon, France) with BSA as standard. Subsequently, equal amounts (50 μg) of protein samples were separated on 12.5% SDS-PAGE and electrotransferred to a nitrocellulose membrane (Pall Corporation, Pensacola, FL, USA). Loading of equal amounts of protein in each line was verified by Ponceau S (Carl Roth, Karlsruhe, Germany) staining. After incubation the membranes overnight at 4°C in blocking solution (5% nonfat dried milk powder), membranes were incubated with primary antibodies against IGF-1 (polyclonal anti-IGF-1 antibody; Santa Cruz Biotechnology, Inc., Santa Cruz, Ca, USA) and Vinculin (monoclonal anti-Vinculin antibody; Invitrogen Corporation, CA, USA) as a reference protein for normalization. The membranes were washed, and then incubated with a horseradish peroxidase conjugated secondary polyclonal anti-rabbit-IgG antibody (Sigma-Aldrich, Steinheim, Germany) at RT. Afterwards blots were developed using ECL Plus or ECL Advanced (both GE Healthcare, Munich, Germany). The signal intensities of specific bands were detected with a Bio-Imaging system (Syngene, Cambridge, UK) and quantified using Syngene GeneTools software (nonlinear dynamics).

### Statistical analysis

Values presented in the text are means ± SD. Data were analyzed by Student’s t test with dietary carnitine concentration as factor using the Minitab statistical software (Release 13, Minitab Inc., State College, PA, USA). Means were considered significantly different at P < 0.05. P-values of microarray data have been corrected for multiple testing by the Benjamini and Hochberg adjustment method. miRNAs with an adjusted P-value < 0.05 were considered to be differentially expressed miRNAs by carnitine supplementation. Subsequently, genes found to be significant by the one-way ANOVA test have been subjected to the Tukey's ‘Honest Significant Difference’ test to determine which groups contribute most to the significant difference.

## Availability of supporting data

The microarray data set supporting the results of this article is available in the Gene Expression Omnibus repository, GEO Series accession number GSE58537, http://www.ncbi.nlm.nih.gov/geo/query/acc.cgi?acc=GSE58537.

## Electronic supplementary material

Additional file 1: **Differentially expressed miRNAs by supplemental carnitine in skeletal muscle of obese Zucker rats.** Spreadsheet contains all differentially expressed miRNAs by carnitine with an adjusted P-value < 0.05. (XLSX 24 KB)

Additional file 2: **Predicted target genes of the 17 validated, most differentially expressed miRNAs by supplemental carnitine in skeletal muscle of obese Zucker rats.** Spreadsheet contains target genes of the validated 11 up- and 6 down-regulated miRNAs by supplemental carnitine predicted by at least one and a maximum of three online free available algorithms TargetScan, miRanda and miRDB. (DOCX 21 KB)

## Abbreviations

miRNA: microRNA
qRT-PCR: Quantitative real time-polymerase chain reaction
IGF-1: Insulin like growth factor 1
GO: Gene ontology
KEGG: Kyoto encyclopedia of genes and genomes.
