# Supplementary material for: Supplemental carnitine affects the microRNA expression profile in skeletal muscle of obese Zucker rats
Source: BMC Genomics. 2014 Jun 21;15(1):512. doi: 10.1186/1471-2164-15-512 (PMC4078242; doi:10.1186/1471-2164-15-512)
Supplement: Supplementary file 2 — Additional file 2: Predicted target genes of the 17 validated, most differentially expressed miRNAs by supplemental carnitine in skeletal muscle of obese Zucker rats. Spreadsheet contains target genes of the validated 11 up- and 6 down-regulated miRNAs by supplemental carnitine predicted by at least one and a maximum of three online free available algorithms TargetScan, miRanda and miRDB. (DOCX 21 KB) [file 12864_2013_6173_MOESM2_ESM.docx]

Table S2 Predicted target genes of the 17 validated, most differentially expressed miRNAs by carnitine in skeletal muscle of Zucker rats

| **miRNA** | **Predicted target genes** |
| --- | --- |
| ***Up-regulated*** | |
| rno-miR-10b-5p | GPM6A, WNT5A |
| rno-miR-208a-5p | ACTN4, ADARB1, AFAP1L1, AFF4, ALDH9A1, AMN1, ANO1, ANP32B, APLN, ARHGAP32, ARIH1, ARMC1, ARPP21, ATF6, B3GALNT1, BCL2L2, BCOR, BECN1, BICD2, BMPER, BNIP3L, BPNT1, BTG3, CAMK2B, CAR2, CATSPER2, CCL25, CCND1, CCNJ, CCNYL1, CD36, CDH2, CDK10, CDK19, CEP76, CERS1, CLIC5, CLTC, CLVS1, CML1, CNKSR2, CNOT8, COX11, CPSF4, CREBZF, CRELD2, CRYBG3, CSTF2, CSTF2T, CTNNA1, CTR9, CUL3, CWF19L2, CYFIP2, DARS2, DDHD1, DEGS1, DENND5A, DGKB, DHX36, DIO1, DNAJC5G, DNER, DSN1, DUOX2, DYNLT3, EIF5, ELAC1, ELAVL2, ELF5, EMP2, ENOPH1, EPB41L4A, ERAP1, ETV1, FAM108B1, FAM115E, FAM118B, FAM168A, FAM18B2, FAM70A, FGFR1OP2, FOXI1, FOXN3, FRY, FUT4, GABRA1, GALNT4, GDF1, GFI1, GGNBP1, GINS3, GLS, GLTSCR1, GLUD1, GPR116, GPR155, GPR19, GPR85, GPX5, GRB10, GREM2, GRP, GSN, HAVCR2, HDDC3, HERC2, HEXB, HMGCR, HMGN3, HNRPH1, HOXC4, HP1BP3, HSPH1, IDH2, IER5, IGF1, ING4, IPMK, IPO9, IRF6, ISPD, ITPR2, JAKMIP3, JAZF1, KCTD4, KDM5B, KLF7, KLHL23, LACE1, LATS2, LCP1, LECT1, LRAT, LRP1B, LRP2, LRRC4C, MAFG, MAN1A1, MAP4K5, MARCH8, ME1, MGAT4A, MID1IP1, MIER1, MILL1, MKNK1, MKRN2, MPPED1, MRPL52, MSL2, MTF1, MTFMT, MTMR6, MUM1L1, MXI1, N5, NAA15, NEUROD6, NKAP, NKRF, NOG, NOVA1, NSG1, NUDCD1, NUP54, NUSAP1, NXT2, OBP3, ONECUT1, OTX2, P2RX5, P2RY10,, PAFAH1B1, PAIP2, PALMD, PARN, PCDH19, PCDHGA1, PCDHGA10, PCDHGA11, PCDHGA12, PCDHGA2, PCDHGA3, PCDHGA5, PCDHGA7, PCDHGA9, PCDHGB7, PCDHGC3, PDCL, PEPD, PHF21A, PIGH, PIGM, PIWIL2, PLA2G12A, PLEKHA1, PLEKHA3  PNPT1, POLD3, POLE3, PON2, PPAP2B, PPP1R3D, PRC1, PRKRA, PRL2B1, PRPF18, PRRX2, PTGIS, PTPN11, PTPN20, PTPRT, PVRL4, RAB33B, RAB9B, RABGGTB, RAD23B  RALBP1, RALYL, RAP1A, RDX, REM1, RFX7, RHOQ,, RNASEH2A, RNF11, RNF111, RNF38, ROCK2, RORA, RPESP, RREB1, RRN3, SAR1A, SAR1B, SCAMP1, SCG5, SCOC, SEC63, SERINC1, SEP 12, SIAH1A, SLC12A3, SLC12A6, SLC16A4, SLC1A2, SLC23A2, SLC24A2, SLC35E3, SLC40A1, SLC44A1, SLC7A11, SMAD7, SMARCA2, SMC5, SNX16, SOS2, SPARC, SPAST, SPCS2, SRPK2, SRSF1, SRSF11, ST3GAL5, STAG1, STAU2, STC1, STXBP1, TAB2, TBX18, TCEAL1, TCF7, TEAD3, TES, TEX10, TFB1M, TFPI, THBS2, TLK2, TM4SF20, TMEM130, TRA2A, TRAPPC2, TRAPPC8, TRIL, TROVE2, TRPM7, TSC22D2, TSPAN13, TSPAN5, TUBA1B, UBE2D4, UBE2K, UBE2N, UBE2V2, UFC1, UNC119B, USP24, USP26, USP40, USP47, USP49, USPL1, VHL, VPS13A, WBSCR16, WLS, WNT9A, ZBTB1, ZBTB20, ZFAND6, ZFP287, ZFP317, ZFP322A, ZFP365, ZFP36L1, ZFP512, ZFR |
| rno-miR-223-3p | ACSL3, DDIT4, F3, IGF1R, MTPN, RNF34, RPS6KB1, STK39, UBE2A |
| rno-miR-434-3p | ALCAM, CYP26B1, DAZAP1, LRRC56, MORF4L2 |
| rno-miR-190a-5p | ARPC5, CDKN1B, NLGN1, TBC1D14 |
| rno-miR-30d-5p | ACTC1, ADRA1D, ANKRA2, ARID4B, ARL6IP6, B4GALT6, BECN1, BTBD10, CADPS, CALCR, CALU, CAPN7, CBFB, CDC37L1, CHKA, DDIT4, EPB41L3, FAM43A, FOXG1, GABRB1, GALNT3, GNAI2, H2AFY, IDH1, JOSD1, KCNA4, KCNJ12, LHX8, LPPR4, MCF2L, MMD, NDEL1, NEFL, NT5E, OMG, PAWR, PDCL, PER2, PGM1, PON2, PPARGC1B, PTP4A1, PTPN2, RAB15, RAB38, RHEBL1, RNF2, RRAD, SCN8A, SERPINE1, SIAH2, SLC30A4, SLC5A3, SLC7A10, SNAI1, SNX10, STK39, TIA1, TTPA, TWF1, UBE2I, UBE2V2, UNC5C, VAPA, WIPF1, YPEL5, YWHAZ, ZDHHC20 |
| rno-miR-347 | FADS1 |
| rno-miR-493-5p | ABCB10, ABCB1A, ADAM10, AHDC1, AIG1, ALG14, ANKRD17, ANO4, AP1AR, AP1S3, AP3S1, APLP2, APOO, ARFGEF1, ARHGAP12, ARL13B, ARL15, ARMC10, ATAD2, ATF7, ATL2, ATP2A2, ATP5A1, BCOR, BHLHE22, BTAF1, BTBD3, BTG1, CADPS2, CALB1, CAMSAP2, CASC4, CCDC165, CCDC169, CCNG1, CCNG2, CCNT2, CCRN4L, CDC14A, CDH11, CDH2, CDH6, CENPJ, CENPW, CHMP1B, CITED2, CKS2, CLDN23, CNKSR3, COL11A1, CPNE8, CREM, CRISPLD2, CRYZ, CSGALNACT2, CSN2, CSPP1, CTDSPL, CTNND2, CTTNBP2, CXCL3, CXCR4, CYYR1, DAB2IP, DCLK3, DDX3X, DGKB, DHTKD1, DHX36, DIP2C, DKK2, DLD, DLL1, DNAJC14, DNAJC21, DSC2, DYRK2, EEA1, EGFEM1, EIF4ENIF1, ENTPD6, ERAP1, ERO1L, ESRP2, FAM120A, FAM179B, FAM204A, FAM35A, FAM55C, FAM69A, FAM76B, FAT1, FBRSL1, FBXL22, FBXO9, FBXW11, FEM1C, FERMT2, FGFR3, FUBP1, FYB, GABRA5, GAD1, GIN1, GINS3, GIPR, GLI4, GLS, GMCL1, GPC4, GPRC5B, GPS2, GRINL1A, GTF2IRD1, GTF3C3, HAO2, HERPUD2, HHIP, HIAT1, HIBCH, HIF1A, HIPK1, HIST1H2BH, HIVEP2, HMGCS1, HNRNPA0, HNRNPA3, HOMER1, HOOK3, HP1BP3, HRASLS, HYAL4, IFRD1, IRX2, IRX3, ITGB1, ITSN2, JAKMIP2, JPH3, JUN, KAT6A, KBTBD2, KCND2, KCNJ6, KCTD4, KDELR2, KDM1B, KDM5B, KDM6A, KLF3, LACTB2, LIMCH1, LIX1L, LMBR1, LRP12, LRRTM3, LSM14A, LY49I4, LY49S3, LY49S4, MAMDC2, MAP1B, MARCH3, MARCKS, MARK1, MBNL2, MED17, MED26, MEF2C, MFSD6, MILR1, MLLT10, MMACHC, MPHOSPH9, MRPL20, MRPL42, MSMO1, MTDH, MUM1L1, MYCT1, NAB1, NAV2, NCOA1, NDUFS5, NEO1, NIPA2, NKX6-1, NLGN3, NR1D2, NRCAM, NUDCD1, NUDT12, NUDT19, NUDT4, NXPH1, OAS1H, OMG, OSBPL6, OTUB1, OVOL1, P2RY1, PACSIN2, PAIP1, PARD6B, PCDHA1, PCDHA10, PCDHA11, PCDHA12, PCDHA13, PCDHA2, PCDHA3, PCDHA4, PCDHA5, PCDHA6, PCDHA7, PCDHA8, PCDHA9, PCDHAC1, PCDHAC2, PCMTD2, PCNX, PFN2, PGRMC2, PHF2, PHF20, PIAS1, PIGH, PITPNB, PKN2, PMP22, PPAP2A, PPM1E, PPP2R5D, PPP3CA, PPTC7, PRKD3, PRPF38B, PRRG1, PSD2, PTDSS1, PTGES3, PTGES3L1, PTPN12, PUM2, RAB33B, RAPGEF4, RASAL2, RASL11B, RBM34, RCBTB1, REEP1, RFX7, RNF111, RNF138, RNF144B, RPE, RRP15, SALL1, SAT1, SATB2, SBNO1, SEC24B, SEC63, SELS, SEMA5A, SF3B1, SGPP1, SH2B3, SH2D3C, SIN3A, SIPA1L2, SKIV2L2, SLC22A23, SLC25A32, SLC25A37, SLC26A3, SLC34A2, SLC35B3, SLCO1A4, SMC6, SNAI1, SNAP23, SORCS3, SOS2, SP3, SPRYD3, SPTAN1, SRD5A3, SRPK2, SRPX, SRSF11, SRSF3, ST6GAL1, STAM, STK4, SULT1C2A, SUV39H2, SV2B, SYNM, SYT4, TAP1, TBC1D24, TBC1D8, TBK1, TBL1XR1, TBX18, TCF7L2, TFAP2E, TGFBR1, TGFBRAP1, TJP1, TM2D3, TMCO3, TMED7, TMEFF1, TMEM106B, TMEM164, TMEM204, TMEM68, TMPRSS15, TMPRSS2, TMX1, TNRC6B, TRDMT1, TRHDE, TSC22D2, TSPAN1, TTC7B, UBE2V2, UBE3A, UBR7, UNC50, USP32, USP45, VAMP2, VASH2, VOM2R18, VOM2R31, VPS54, VSTM4, WDR33, YIPF6, YTHDF3, ZBTB11, ZC3H14, ZCCHC2, ZFAND5, ZFP157, ZFP37, ZFP384, ZFP385B, ZFP518A, ZFP706, ZFP804A, ZFP84, ZFX, ZIC2, ZMYND19, ZYG11B |
| rno-miR-29a-5p | ACBD6, ACSL4, ALG13, AP2B1, AQP4, ARID4B, ARL6IP6, ATP6V1C1, AZIN1, BCL2L14, BNIP3, CALCR, CAST, CBLB, CCNG1, CCR5, CCT2, CDC37L1, COL4A4, COMMD10, COQ10B, CRBN, CTSE, DCLK1, DDI2, DDX19A, DONSON, DPYS, DUSP11, EIF4A2, ELOVL6, ESM1, FBXO46, GATA4, GNG5, GPR56, GPR64, GRAMD3, GRID2, HAVCR1, HIVEP2, IDH1, IK, ITM2B, KCNK1, KCNQ3, KLHL24, LETM1, LOX, LPAR1, LPPR4, MAPK6, MCCC2, MFAP3L, MOAP1, MOCS2, MPP4, NAGA, NID67, NR4A2, NR4A3, NSDHL, OBP1F, OLFM3, OPTN, PA2G4, PAWR, PCGF6, PDCL, PGRMC1, PLA2G7, PPM1A, PRKG2, PRLHR, RAB21, RAB8A, RALBP1, RCAN1, ROD1, ROGDI, SCN9A, SLC12A2, SLC16A7, SLC46A3, TRAK2, TRAM1, UNC5C, WDR89, ZBTB25 |
| rno-miR-451-5p | CAB39, FIGN, OSR1, SAMD4B |
| rno-miR-190b-5p | ARPC5, CDKN1B, NEUROD1, NLGN1, PHLPP1, TBC1D14, WSB1 |
| ***Down-regulated*** | |
| rno-miR-21-3p | ABHD6, ACSL4, ADRA2B, AP1B1, AP3M1, ARL8B, ARPC5, ASAH1, ATAD1, ATF3, ATG4B, ATP6AP1, BECN1, CALB1, CALHM2, CAMKK2, CAPN7, CAPZA2, CEP55, CHMP4C, CHP, CLDN11, CLTC, CNKSR2, COL3A1, CRIPT, CTHRC1, CXADR, CXXC5, CYP26B1, CYP51, DIXDC1, DYNLT3, ECHDC1, ELAVL2, ERRFI1, FAM107B, FAM126B, FAM174A, FGF13, FMC1, FMO2, GLRX1, GNAO1, HBP1, HNRNPA1, HNRNPK, IGSF11, IL6ST, INPP1, ITPR1, KHDRBS2, KLHL7, LCP2, LKAP, MARCH3, MATR3, MCART1, MESDC2, MRPL47, MS4A2, MS4A6B, MTDH, MYNN, MYOCD, NANP, NAP1L3, NECAP1, NELF, NOX4, NPC2, NR3C1, OLAH, PAF1, PCDHA1, PCDHA10, PCDHA11, PCDHA12, PCDHA13, PCDHA2, PCDHA3, PCDHA4, PCDHA5, PCDHA6, PCDHA7, PCDHA8, PCDHAC1, PCDHAC2, PDAP1, PDHB, PPIG, PPP1R3C, PRPF4B, PSG19, PTBP2, PTF1A, PTMA, PTP4A1, QRSL1, RAB7A, RAB8A, RCAN1, REG3A, RHOBTB2, RMND5B, RNASE9, SCOC, SDHD, SERPINI1, SFXN1, SHANK2, SKAP2, SLC18A2, SLC7A3, SMCP, SORBS2, SRGN, STRA6, TDG, TESK2, TFG, TGFA, THBD, TMEM43, TRAM1, UBE2I, UBQLN1, UNG, VAPA, ZC3H14, ZFP207, ZKSCAN1 |
| rno-miR-3596c | ACSL3, ADRB2, AFF4, ANKRD13C, ARHGAP12, CHMP5, CHP, CSPP1, CUL1, DAG1, EIF4A1, EIF4ENIF1, EP300, FAM76B, FMR1, GABRA1, GIN1, GPR116, HACE1, HMGB2, JAG1, JAZF1, LSM14A, MAPK6, MARCH6, MARK1, MCFD2, MED14, MEF2C, MIER3, NR3C1, NUP153, PACSIN2, PAPOLG, PDS5B, PITX1, PRKRIR, PUM1, RANBP2, RB1CC1, REV3L, RIF1, SELK, SLC19A2, SPOCK3, STRBP, TAB2, TMCC1, TSHZ3, UBE2D1, UBL3, USP9X, VCAN, VPS37A, ZBTB2, ZFP292, ZFP652, ZFPM2 |
| rno-miR-3584-3p | SALL3 |
| rno-miR-466b-1-3p | AASS, ABI2, ABLIM3, ACNAT2, ACSL4, ADARB1, ADCK1, ADI1, AP3S1, API5, APOOL, AQP3, ARG1, ARL6IP6, ARPC5L, ATP11A, ATPAF1, BCL6, BCOR, BHLHE41, BIRC6, BMF, BMP5, BRMS1L, BTAF1, CAB39, CASK, CBLL1, CCDC12, CCDC169, CCDC68, CCDC82, CCL11, CCNI, CCNT2, CD164, CDH1, CDH11, CDH2, CENPI, CENPW, CES1F, CHEK1, CHMP4C, CLASP2, CLDN8, CLSPN, CLTC, CML1, CNOT6L, CNR1, COMMD2, CREM, CSNK1G3, CUL3, CWC25, CYP2J10, DAAM1, DIAPH3, DMRTC2, DNAJB1, DNAJC14, E2F6, ENPP4, EPB41L1, ERGIC1, ETNK1, EXOC8, FAM104A, FAM164A, FAM168A, FAM19A4, FAM60A, FAM91A1, FBXO30, FGFR2, FUT4, GABPB1, GABRA2, GABRB3, GAD1, GHR, GIMAP8, GK, GLB1L2, GMNN, GOLPH3, GPCPD1, GPR4, GRM1, GTF2B, GTSE1, GUCY1B3, GXYLT1, HAUS8, HIVEP1, HMGCR, HNRNPU, HOXD1, HSD17B12, HTR2C, IER3, IGF1, IL2, IRF9, ITPR1, JAG1, JUN, KANK4, KAT6A, KBTBD2, KCTD4, KHDRBS1, KIF11, KIF1B, KIF23, KIF2A, KIT, KLF15, KLF3, KLHL28, KLHL31, LAP3, LARP1B, LARP5, LEMD3, LHFPL2, LIN54, LPAR1, LRRC8C, LRRFIP1, LRRTM2, LRRTM3, MAP3K4, MAP4K3, MARCH5, MED17, MFSD2A, MIER3, MIS18A, MTF1, MTMR3, MXD1, NAP1L3, NAT13, NCOA1, NDRG4, NECAP1, NFATC3, NPAT, NR3C2, NRG1, NUPL1, NXT2, OAS3, OSGEPL1, PAPOLG, PCK1, PDCD6IP, PDE7A, PHEX, PITPNB, PKD2, PLK4, POGZ, PPP1R15B, PPP1R2, PPP4R2, PRKCB, PRKCD, PSMC6, PSPC1, PTBP2, PTPRA, RAB11A, RABGAP1, RAPGEF4, RASSF1, RBFOX1, RBM24, RBM41, RBM7, RERG, RGS4, RHOQ, RHPN2, RIPK1, RNGTT, RPGRIP1L, RPS6KA5, RSBN1, SATB2, SBK2, SECISBP2L, SELE, SELT, SERF2, SETD5, SIX4, SIX6, SLA, SLC21A4, SLC25A13, SLC26A3, SLC2A12, SLC30A10, SLC38A2, SMARCA5, SMURF2, SNAP29, SNX19, SNX27, SNX5, SOX9, SPAG6, SPZ1, SRPK2, SRSF3, STAG2, STXBP1, STXBP3, SVS3B, SYNJ2BP, SYNPR, TCF7L2, TMED5, TMEFF2, TMEM161B, TMEM68, TMOD3, TMPRSS2, TOPBP1, TRAM1L1, TRIB2, TRIM42, TSC22D2, TSPAN2, TSPAN3, TYMS, UBA6, UBAP2, UBE3A, UBTD2, UGT8, UNC13C, UPP2, UQCRC2, USP53, VSTM2B, WDR43, WIF1, XPO4, YLPM1, ZBTB44, ZC3H7A, ZCCHC2, ZDHHC21, ZFAND5, ZFP110, ZFP26, ZFP329, ZFP36L2, ZFP462, ZFP644, ZFP804A, ZFP91, ZMIZ1 |
| rno-miR-883-5p | ABCG1, ACSF2, ADCK3, ADM, ADM2, AFF4, ALDH1A7, ARHGAP9, ATPAF1, BHLHE23, CCL24, CCL28, CDH17, CLDN19, CNDP1, CNPY4, CNTF, CPN2, CUL3, CYB561D1, DCAF11, DCX, DDAH1, DDX19A, ECEL1, ELK4, EMP2, ERI2, FAM111A, FAM178A, FAM46C, FAM64A, FAT4, FNBP4, GABRB3, GOLIM4, GOSR1, GPNMB, GPX5, GTF2I, HEBP1, HEBP2, HHIP, HYOU1, ITGB1, ITK, JRK, KDM5B, KEG1, KIF5A, KLHL14, LCE1M, LKAP, LRRC39, MAGEA11, MAP1LC3B, MARCH6, MEGF8, MEIS2, MKNK2, MLLT4, NKAIN1, NOL4, PADI2, PAPSS2, PCSK7, PGAM5, PIK3CA, PLP1, POP7, PPHLN1, PPP1R1C, PTRH2, RAB6A, RABIF, RARG, RASGRF1, RBM14, RNF217, RPL15, RPS6KB1, SEP 05, RRAGB, SNX30, SNX4, SPOP, ST3GAL2, SUMO1, SWAP70, SYN2, TACC1, TAPT1, TBC1D5, TEF, TGFA, TM4SF1, TMEM196, TMEM48, TMEM50A, TMEM68, TRPS1, TSPAN18, UHMK1, UPF2, USP3, VHL, VIPR2, VPS29, WDTC1, YIPF3, ZBTB25, ZFP316, ZFP324, ZFP426, ZWILCH |
| rno-miR-466c-5p | H3F3B, SLC6A8, UBE2N |

Rno: *Rattus norvegicus*.

Predicted target genes for differentially expressed miRNAs was performed by using three online free available algorithms TargetScan release version 6.2, miRanda and miRDB.
